# Polyoxometalate Etching of NMO@NF for Highly Efficient Oxygen Evolution Reaction in Water Splitting

**DOI:** 10.3390/ijms26073107

**Published:** 2025-03-28

**Authors:** Ting Chen, Xiang Han, Zefen Wang, Chaoying Li, Mei Li, Xiongdiao Lan, Yingying Ning, Jingxin Wang, Pengru Liu

**Affiliations:** 1 Guangxi Key Laboratory for Polysaccharide Materials and Modifications, School of Chemistry and Chemical Engineering, Guangxi Minzu University, Nanning 530006, China; ct20241001@163.com (T.C.); hanxiang202303@163.com (X.H.); lzy120130lzy@163.com (C.L.); emilyve656@163.com (M.L.); ningying0408@163.com (Y.N.); 13393929075@163.com (J.W.); 2 Guangxi Autonomous Region Center for Analysis and Test Research, Nanning 530022, China; wangzefen@126.com

**Keywords:** wet chemical etching, nickel molybdate, cationic defect, oxygen evolution reaction, electrocatalysis

## Abstract

In this study, PTA&PMA/NiMoO_4_@NF was synthesized on nickel foam through wet chemical etching to promote the kinetics of the oxygen evolution reaction (OER) effectively. OER benefits from two cationic (Ni and Mo) defects and the optimized electronic configuration of PTA&PMA/NiMoO_4_@NF. Thus, it only needs 200 mV to reach the current density of 10 mA cm^−2^ in 1.0 mol/L of KOH. This value is nearly 100 mV lower than the value needed by pure NiMoO_4_. After being used as an anode for water splitting in an alkaline solution, the as-obtained catalyst can operate at a current density of 10 mA cm^−2^ for 24 h of good stability. The synthesis strategy adopted in this study can provide an effective, low-cost, simple, and convenient strategy for improving the OER electrocatalytic performance of other transition metal oxides.

## 1. Introduction

Electrocatalytic water splitting (2H_2_O → O_2_ + 2H_2_), which is an efficient and sustainable method for hydrogen production, holds promise for addressing the energy crisis and environmental issues caused by traditional fossil fuels [1]. Water electrolysis typically consists of the hydrogen evolution reaction (HER) and the oxygen evolution reaction (OER). HER is a two-electron proton coupling reaction, whereas OER is a four-electron proton coupling reaction, requiring high energy to overcome the OER barrier. It can significantly reduce the efficiency of the electrochemical water splitting reaction. Therefore, OER is generally considered the decisive step in the water splitting reaction. Selecting an appropriate catalyst is crucial to overcome the impact of the OER process on the electrochemical water splitting process [2]. Hence, developing high-performance electrocatalysts that accelerate OER kinetics is urgently needed.

Currently, the most commonly used anode catalysts are noble metal oxides, such as RuO_2_ and IrO_2_. However, their high cost and low reserves hinder their practical application [3,4]. Transition metal oxides are low-cost, have high reserves, and exhibit good OER catalytic activity [5,6,7,8]. Among the transition metal-based compounds, binary nickel-based materials, particularly NiMoO_4_ (NMO), have been extensively developed as efficient water-splitting electrocatalysts; at present, NMO is the most studied nickel-based electrocatalyst. Although single-metal oxides such as Co_3_O_4_ [9], Fe_2_O_3_ [10], and MnO_2_ [11] have been widely explored for similar applications, their performance is often constrained by limited active sites, poor electrical conductivity, or instability under operational conditions. In contrast, the dual-metal composition of NiMoO_4_ addresses these limitations through its complementary functionalities: Ni atoms contribute high intrinsic redox activity and facilitate charge transfer, and Mo atoms enhance structural stability and modulate the electronic states to optimize intermediate adsorption/desorption kinetics [12,13]. However, NMO electrodes are still affected by poor electronic conductivity, insufficient ion transport and diffusion, and structural instability during long-term cycling. Therefore, fabricating NMO with controlled nanostructures and designing reasonable structural engineering are important but challenging. In particular, various low-dimensional NMO nanostructures directly grown on conductive current collector substrates (e.g., Ni/Cu foam [14], graphene [15], and carbon substrates [16]) reduce charge carrier scattering at grain boundaries and are easily integrated into flexible devices with certain specific applications. These nanostructures are particularly preferred for directional electron transport. Various NMO/carbon composites have been synthesized to overcome the poor conductivity of pure NMO by combining NMO nanostructures with graphene [17], carbon nanotubes [18], conductive polymers [19], and porous carbon structures [20,21]. In addition to carbon composites, several heteroatoms (e.g., Mn [22,23], P [24], Zn [25], Ce [26]) have been doped into NMO, or oxygen vacancies have been generated in the NMO lattice [27]. Furthermore, NMO has been combined with other metal oxides [28,29,30,31,32,33] or sulfides [34,35,36] to form heterostructure electrodes and improve electrochemical performance. However, the reported composite or doping methods of NMO involve multiple complex chemical and physical processes, which have high economic costs or are environmentally unfriendly in the synthesis path.

Research shows that chemical acid etching has become a research hotspot. A multifunctional polyoxometalate (POM; also called heteropoly acid) etching method could clearly reconstruct NiFe layered double hydroxide (LDH), including three-dimensional morphology nanocutting, Fe^3+^ and α-Ni(OH)_2_ active substance reconstruction, and the generation of multiple Ni, Fe, and O vacancies, which subtly coordinate local environments and electronic structures of iron and nickel cations, creating additional active sites for reduction reactions. Moreover, the embedding of POM polyanion clusters can tune the electronic configuration of NiFe LDH to facilitate the formation and transformation of intermediate states, thereby accelerating the electrochemical process [37]. As a result, the high catalytic activity (η_10_ = 206 mV) and excellent stability (negligible η_500_ change over 24 h) of NiFe LDH-PMo_12_ can be achieved [38]. An in-depth understanding of the intrinsic activity origin of POM etching has been gained by combining the theoretical and experimental results, thereby proving the feasibility of POM etching as a promising posttreatment technology. POMs are a class of well-defined transition metal oxide clusters with tunable redox properties and strong acidity. They have been widely explored as molecular catalysts for acid and oxidation reactions.

On the basis of the above statement, highly active NMO@NF was prepared via a low-cost, simple, and convenient wet chemical etching method that used phosphotungstic acid (PTA) and phosphomolybdic acid (PMA). It also offered potential avenues for designing new materials. Experimental characterizations revealed two cationic (Ni and Mo) defects and the optimized electronic configuration of NMO after PTA and PMA etching, which promoted the OER process.

## 2. Results and Discussion

### 2.1. Material Synthesis and Characterization

**Figure 1:**
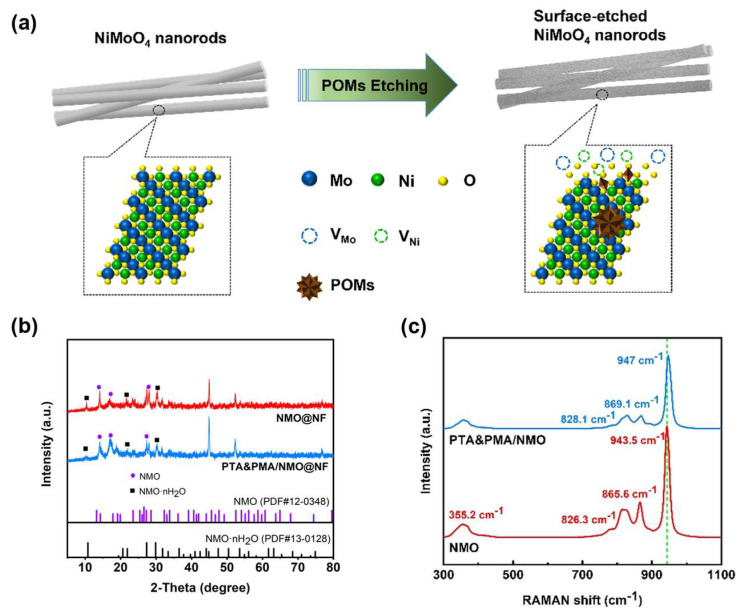
Structure and phase composition of the catalysts. (**a**) Diagram of the synthesis process for PTA&PMA/NMO@NF; (**b**) XRD pattern Raman spectra of the as-prepared NMO@NF and PTA&PMA/NMO@NF; and (**c**) Raman spectra of the as-prepared NMO and PTA&PMA/NMO.

The electrocatalytic samples of PTA&PMA/NMO@NF are prepared using a simple hydrothermal synthesis method and the wet chemical etching method. A mixed solution of ammonium molybdate and nickel molybdate is prepared, and NMO@NF is synthesized in situ on the NF using the hydrothermal method. Subsequently, a weakly acidic mixed solution of PTA and PMA is prepared. This solution easily etches the surface of NMO@NF, thereby obtaining a PTA&PMA/NMO@NF electrocatalyst. The etching schematic diagram is shown in Figure 1a.

The atomic ratio is obtained via the ICP test, during which the contents of Ni, Mo, P, and W cations are detected in the NMO and PTA&PMA/NMO electrocatalysts. After POM etching, two cationic (Ni and Mo) defects are produced in NMO, and a small number of P and W atoms are attached to PTA&PMA/NMO during the etching process (Table S1). Additionally, the Ni/Mo element ratio in NMO is approximately 5:8, whereas that in PTA&PMA/NMO is approximately 5:9. In particular, the P and W element contents in PTA&PMA/NMO are approximately 280.7 μg/g and 67.3 μg/g, respectively. This finding confirms the insertion of POM multianion clusters. The partial decomposition of POMs retains residual P substances by combining the TEM/EDX analysis results, and some n(PO_4_)^3−^ structural building unitsare preserved at the surface of the nanosheets [34].

The XRD analysis results show that for NMO@NF and PTA&PMA/NMO@NF electrodes (Figure 1b), the NF substrate had three strong characteristic peaks at 44.5°, 51.8°, and 76.3°. Figure 1b shows that the crystal phases of NMO·nH_2_O (JCPDS card number: 13-0128) and NMO (JCPDS card number: 12-0348) coexist in NMO@NF after wet chemical etching. These crystals are retained in PTA&PMA/NMO@NF [39]. The peak widths of PTA&PMA/NMO@NF at 14.2°, 16.8°, and 18.9° significantly increase because of the lack of cations and changes in the lattice structure after etching. The lattice constants and volume slightly increase. This phenomenon can be attributed to POMs exhibiting tunable redox properties, superacidic characteristics, and exceptional stability in both solution and solid states. These attributes enable them to replace traditional liquid acids (e.g., HF, HCl, H_2_SO_4_) and molecular catalysts for oxidation reactions [38,40]. Concurrently, H⁺ ions can etch and corrode the catalyst surface to generate abundant defect sites. Additionally, interlayer anion exchange between the POM clusters of heteropoly acids and the intrinsic anions (such as MoO_4_^2−^) contributes to this phenomenon [38]. This result is consistent with the findings from the SEM, TEM, and XPS analysis.

The Raman spectra are displayed in Figure 1c. The Raman spectrum of NMO shows a strong peak at 943.5 cm^−1^ and some low-intensity peaks at 865.6, 826.3, and 355.2 cm^−1^, all of which are Raman characteristic peaks of NMO [41]. The Raman spectrum of NMO/PT-A&PMA shows a strong peak at 947 cm^−1^ and some low-intensity peaks at 869, 828.1, and 355.2 cm^−1^ [42,43,44]. The Raman spectrum of PTA&PMA/NMO shifts to high frequencies and the peak intensity of PTA&PMA/NMO weakens because the lack of cations causes neighboring anions to contract or expand to maintain charge balance, resulting in a blue shift in Raman peaks after POM etching. Additionally, interlayer anion exchange between the POM clusters of heteropoly acids and the intrinsic anions alters the interlayer interactions and spacing, increasing the frequency of intra- and interlayer vibrations. This observation is consistent with the XRD results.

**Figure 2:**
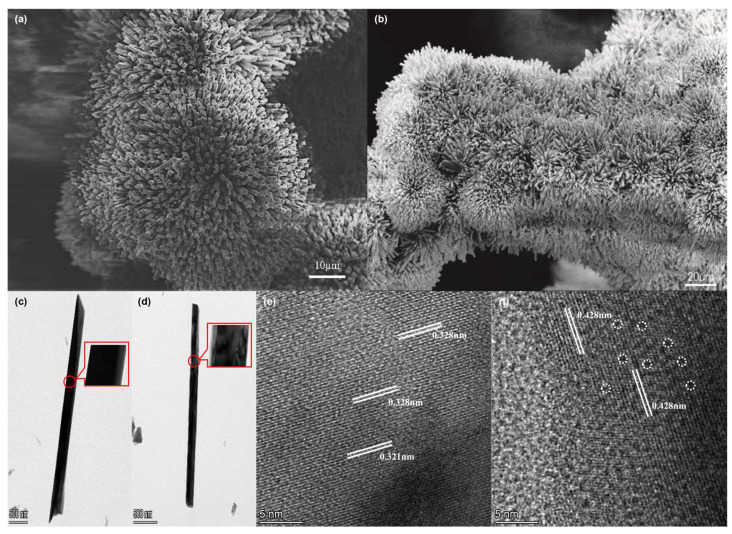
Morphology of catalysts. The SEM images of (**a**) NMO@NF and (**b**) PTA&PMA/NMO@NF. The TEM images of (**c**) NMO and (**d**) PTA&PMA/NMO. The HRTEM images of (**e**) NMO and (**f**) PTA&PMA/NMO.

The morphology and the lattice fringes of samples were characterized by SEM, TEM, and HRTEM. Figure 2a shows that after the hydrothermal synthesis of NMO, NF is almost completely covered by flowerlike clusters composed of NMO nanorods. Although the electrode was platinum-sputtered, the SEM images of NMO@NF showed significant discharging. This finding indicates that NMO has poor conductivity. After chemical etching, the conductivity of PTA&PMA/NMO@NF is significantly improved. This phenomenon may be related to the structural changes caused by the etching [39]. As shown in Figure 2b, the flowerlike clusters are well retained in the PTA&PMA/NMO@NF. In Figure 2c, the surface of NMO is smooth with an average diameter of ~300 nm. After etching, nanorods exhibit a diameter that does not change significantly, but PTA&PMA/NMO (Figure 2d) possesses a surface area that loosens and shows defects because of metal exsolution during the etching process. The HRTEM image in Figure 2e shows that the marked lattice spacing of NMO is 0.321 and 0.328 nm. By contrast, the marked lattice spacing of PTA&PMA/NMO is 0.428 nm. Moreover, some lattice distortion can be found at the interface because of the structure termination due to metal precipitation during etching, as shown in Figure 2f. The element distribution in NMO and PTA&PMA/NMO is shown by EDX elemental mapping (Figure S1), in which all elements are uniformly distributed. The detailed EDX elemental analysis (Table S1) shows that the P/Mo ratio is 1:1.4, which is far higher than the nominal ratio of 1:12 in pure PTA and PMA. This finding indicates that the acid–base reaction between pure PTA and PMA and NMO leads to the partial decomposition of pure PTA and PMA. Moreover, a small number of n(PO_4_)^3−^ structural building units are anchored [45].

**Figure 3:**
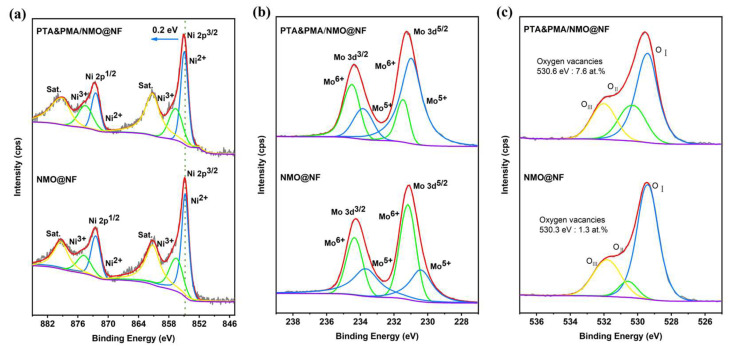
High-resolution XPS spectra of (**a**) Ni 2p, (**b**) Mo 3d, and (**c**) O 1s for NMO@NF and PTA&PMA/NMO@NF.

XPS is used to determine the elemental composition and chemical state of NMO@NF and PTA&PMA/NMO@NF electrocatalytic materials and to understand the changes in the oxidation state of atoms in the NMO catalyst caused by POM etching. The full spectrum shows that Ni, Mo, and O elements are present in NMO@NF and PTA&PMA/NMO@NF (Figure S2). As shown in Figure 3a, the high-resolution Ni 2p spectrum of NMO@NF shows that Ni 2P^1/2^ and Ni 2P^3/2^ doublets are identified at 872.5 and 874.8 eV and at 854.8 and 856.5 eV, respectively. They are also accompanied by two satellite peaks at 861.2 and 879.3 eV [37]. The high-resolution Ni 2p spectrum of PTA&PMA/NMO@NF shows that Ni 2P^1/2^ and Ni 2P^3/2^ doublets are identified at 872.5 and 874.8 eV and at 855 and 856.7eV, respectively. They are also accompanied by two satellite peaks at 861.3 and 879 eV. Unlike the peak position of Ni 2p in NMO@NF, that in PTA&PMA/NMO@NF shifts by 0.2 eV toward a high binding energy. This finding indicates that the valence state of Ni increases and numerous Ni^3+^ exist in PTA&PMA/NMO@NF [46]. The Ni^3+^/Ni^2+^ ratio of PTA&PMA/NMO@NF is 0.77, which is higher than that of NMO@NF in Table S2. This finding suggests that this phenomenon is beneficial for the formation of NiOOH during the OER process [47].

The high-resolution Mo 3d of NMO@NF is shown in Figure 3b, and Mo 3d^3/2^ and Mo 3d^5/2^ doublets are identified at 233.7 and 234.4 eV and at 230.4 and 231.2 eV, respectively. The high-resolution Mo 3d spectrum of PTA&PMA/NMO@NF shows that Mo 3d^3/2^ and Mo 3d^5/2^ doublets are identified at 233.8 and 234.5 eV and 231 and 231.5 eV, respectively. Compared with the intensity of Mo^5+^ NMO@NF, that in PTA&PMA/NMO@NF, which corresponds to the cations (Ni and Mo) in the ICP-OES results, is significantly enhanced. This finding indicates that the oxygen vacancy defect content increases significantly during the etching process. In addition, the O 1s spectrum of NMO@NF (Figure 3c) shows three characteristic peaks at 529.4, 530.6, and 531.8 eV, and the O 1s spectrum of PTA&PMA/NMO@NF shows three characteristic peaks at 529.4, 530.3, and 532 eV [48,49,50]. The characteristic peaks are attributed to metal oxygen (O_I_), relative oxygen vacancies (O_II_), and surface adsorbed oxygen (O_III_). For PTA&PMA/NMO@NF, O_III_ is slightly increased by 0.2 eV; the metal oxygen concentration measured by XPS is 23.7 at.%, which is much higher than that for NMO@NF (17.1 at.%); and the oxygen vacancy concentration measured by XPS is 7.6 at.%, which is much higher than that for NMO@NF (3.1 at.%). The peak density of oxygen vacancies increases significantly; with the introduction of oxygen vacancies, the electronic structure at catalytic sites is effectively modulated to facilitate the formation and transformation of intermediate states, thereby accelerating the electrochemical process [51].

### 2.2. Electrochemical OER Performance 

**Figure 4:**
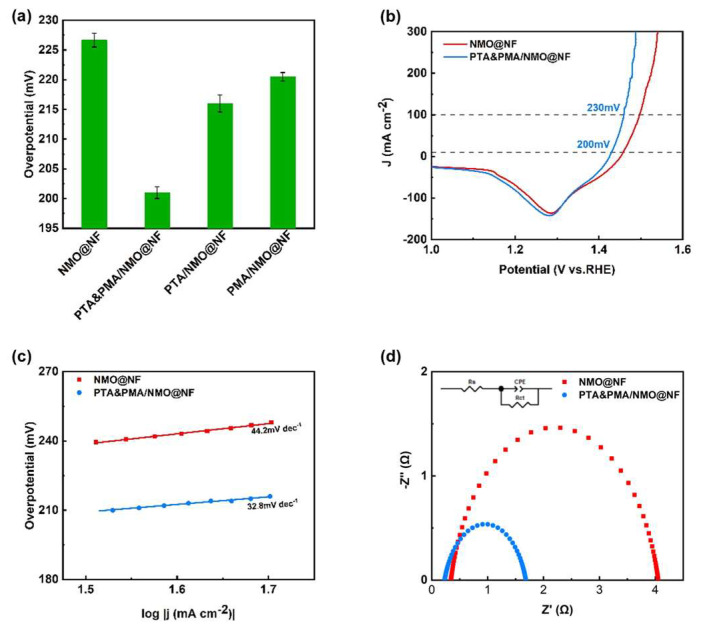
Electrochemical performance tests of the catalysts. (**a**) The overpotential at 10mA cm^−2^ of NMO@NF, PTA&PMA/NMO@NF, PTA/NMO@NF, and PMA/NMO@NF; (**b**) The iR-compensated polarization curves and (**c**) the corresponding Tafel plots of NMO@NF and PTA&PMA/NMO@NF for OER at a scan rate of 5 mV s^−1^. (**d**) Nyquist plots of the electrodes at an overpotential of 10 mV.

OER testing was conducted at room temperature in a 1 M KOH electrolyte solution using a three-electrode system, the electrocatalytic activity of four OER catalysts (NMO@NF, PTA&PMA/NMO@NF, PTA/NMO@NF, and PMA/NMO@NF) for OER was tested. Figure 4a presents a visual comparison of the overpotential at 10mA cm^−2^ among the various catalysts, demonstrating that PTA&PMA/NMO@NF outperforms the others by having the least overpotential. The LSV polarization curves were swept from high potential to low potential to avoid the influence of oxidation current. Figure 4b shows that in the potential range of 1.35–1.28 V, Ni^3+^ was reduced to Ni^2+^, and the overpotential of PTA&PMA/NMO@NF was significantly reduced. As a result, current densities of 10 and 100 mA cm^−2^ were achieved at only 200 and 230 mV, respectively. NMO@NF required overpotentials of 227 and 267 mV to achieve current densities of 10 and 100 mA cm^−2^, while RuO_2_@NF needed an overpotential of 314 mV to afford 100 mA cm^−2^ [43]. ICP-OES and XPS analysis results show that the generation of oxygen vacancies significantly improved the OER performance of the PTA&PMA/NMO@NF electrocatalyst after POM etching because two cationic (Ni and Mo) defects were produced, and the ion leaching behavior in the pristine material may have triggered favorable OH^⁻^ adsorption on the catalyst surface, thereby facilitating electron transfer during the OER process [52,53,54,55]. As shown in the Tafel slope in Figure 4c, the Tafel slope of PTA&PMA/NMO@NF (32.8 mV dec^−1^) was significantly smaller than that of NMO@NF (44.2 mV dec^−1^) and RuO_2_@NF (73 mV dec^−1^) [14]. This finding indicates that the reaction kinetics of PTA&PMA/NMO@NF were better and more favorable for the OER than those of NMO@NF. The kinetics of the OER were studied using EIS. As shown in Figure 4d, the R_ct_ of PTA&PMA/NMO@NF was the lowest (1.5 Ω), which was lower than that of NMO@NF (3.7 Ω). This phenomenon can be attributed to the generation of abundant cationic defect sites and oxygen vacancies on the catalyst surface through chemical etching, which enhances favorable OH^⁻^ adsorption. Consequently, this promotes faster electron transfer and reduces charge transfer resistance [53,54,55]; the PTA&PMA/NMO@NF electrode has better conductivity than NMO@NF, leading to fast electron transfer rates and superior OER performance.

**Figure 5:**
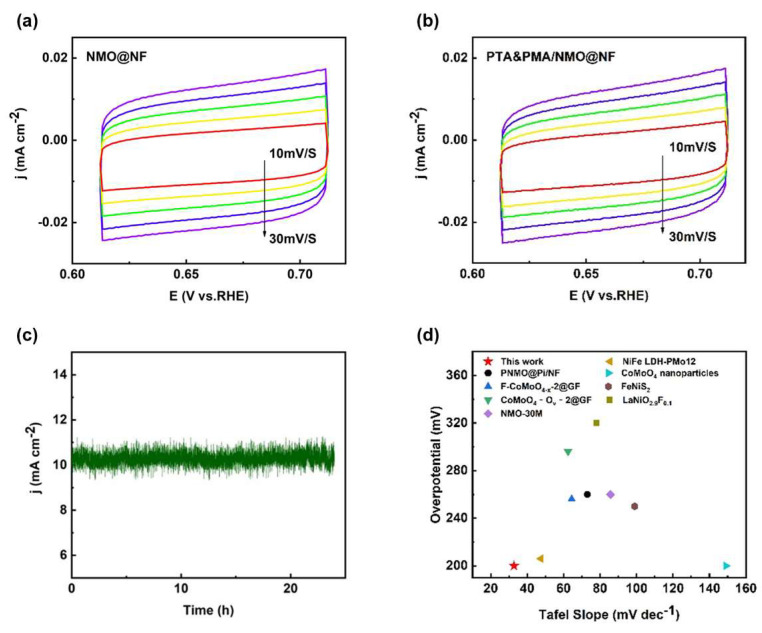
Electrochemical performance tests of the catalysts. (**a**) Comparison of the Rct values for NMO@NF and PTA&PMA/NMO@NF; (**b**) CV curves of NMO@NF/PTA&PMA at different scan rates from 10 mV s^−1^ to 30 mV s^−1^; (**c**) I-t curve of PTA&PMA/NMO@NF at a potential of 1.43 V versus RHE; and (**d**) a comparison chart of different material properties.

The C_dl_ of the catalyst was determined by integrating the CV curves and was found to be proportional to the electrochemically active surface area (ECSA). The capacitance values associated with the double layer (C_dl_) were derived from the CV curves of NMO@NF (Figure 5a) and PTA&PMA/NMO@NF (Figure 5b), which were 1.07 and 1.10 mF cm^−2^, respectively (Figure S3). The Cdl of NMO@NF/PTA&PMA did not show noticeable changes, indicating that its catalytic active surface underwent no significant changes. Catalyst stability had practical significance for its application. The stability of NMO@NF/PTA&PMA was evaluated by the current time method. Figure 5c shows that when the reversible hydrogen potential of NMO@NF/PTA&PMA was 1.43 V and the current density was 10 mA cm^−2^, the curve remained stable after 24 h of the time current stability test, and the change in overpotential was negligible. This finding highlights that NMO@NF/PTA&PMA has outstanding electrochemical stability in the electrochemical process of OER.

**Figure 6:**
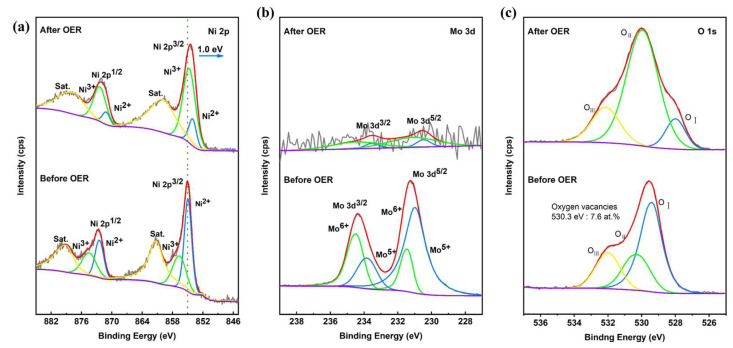
XPS of NMO@NF/PTA&PMA before and after OER. (**a**) Ni 2p, (**b**) Mo 3d, and (**c**) O 1s.

As shown in Figure S4, the SEM image of PTA&PMA/NMO@NF after 24 h of the stability test (PTA&PMA/NMO@NF Sat.) shows that the nanorods covered on the NF did not disappear and were still well preserved. However, the diameter of the nanorods was reduced to approximately 200 nm. The reduction in the nanorod diameter of the PTA&PMA/NMO@NF catalyst after 24 h of stability testing was attributed to the decrease in Mo atoms, as revealed by the ICP-OES and EDS results. Notably, Mo atoms in the catalyst were almost entirely depleted after 24 h of stability testing. Therefore, no significant further changes in nanorod diameter would occur in catalysts subjected to stability tests exceeding 24 h. The transmission electron microscopy results (Figure S5) also confirmed this result. The HRTEM image in Figure S6 shows that the lattice fringes of PTA&PMA/NMO@NF Sat. were not obvious, possibly because of the significant reduction in the content of the Mo element. This observation is in good agreement with the results of ICP-OES (Table S1) and TEM-EDX (Table S3). The element distribution in PTA&PMA/NMO@NF Sat. was shown by the EDX element mapping (Figure S7), in which all elements were evenly distributed. Compared with the XRD spectra of PTA&PMA/NMO@NF Sat. and PTA&PMA/NMO@NF (Figure S8), the peak positions were unchanged. The peak widths of the diffraction peaks of PTA&PMA/NMO@NF Sat. at 14.2°, 16.8°, and 18.9° increased significantly because of the lack of cations and the change in lattice structure after etching. The lattice constant and volume increased slightly. This phenomenon can be attributed to the substantial reduction in the Mo element [47]. These results further demonstrate that the PTA&PMA/NMO@NF electrode has excellent long-term stability. In the Raman spectra of PTA&PMA/NMO@NF Sat. (Figure S9), the peaks belonging to Mo-O and Mo-O-Ni disappearred because of the reduction in Mo atoms. As shown in Figure 6a, the XPS results of PTA&PMA/NMO@NF before and after OER show that after OER, the peak position of Ni 2p moved 1.0 eV to the direction with low binding energy. This observation can be attributed to the decrease in the concentration of Mo atoms [47]. The Ni^3+^/Ni^2+^ ratio was 4.5, indicating that the valence state of Ni increased. This finding suggests that this phenomenon is beneficial to the formation of NiOOH during the OER process. The high-resolution Mo 3d of PTA&PMA/NMO@NF Sat. is shown in Figure 6b. Given the surface reconstruction, the intensity of Mo after OER was weaker than that before OER. The O 1s spectrum of PTA&PMA/NMO@NF Sat. (Figure 6c) showed three characteristic peaks at 528, 530, and 532.1 eV, which were attributed to O_I_, O_II_, and O_III_, respectively. The oxygen vacancy concentration was 18.4 at.%, and the peak density of oxygen vacancy density increased significantly. The metal oxygen concentration was 2.95 at.%, and the peak density of metal oxygen density decreased significantly. The peak position of metallic oxygen moved 1.4 eV toward the low binding energy direction. This phenomenon can be attributed to the reduction in Mo atoms [47].

Additionally, the OER performance of our catalyst was comprehensively compared with the OER performance in the existing literature [1,38,39,42,56,57,58,59] and that of commercial catalysts. The results showed that the OER efficiency of PTA&PMA/NMO@NF surpassed many previously described transition metal electrocatalysts (Figure 5d and Table S4), thereby making it a strong competitor with noble metal-based OER catalysts. The slight change in the overpotential of PTA&PMA/NMO@NF indicated good electrocatalytic performance. By contrast, the reduced Tafel slope indicated that the rate-limiting step was close to the end of the multielectron transfer process. This observation emphasizes the adaptability of the electrocatalyst.

## 3. Materials and Methods

### 3.1. Material

Nickel foam (NF; size of 20 × 30 cm, thickness of 1 mm) was purchased from Kunshan XZH Electronic Materials Co., Ltd. (Suzhou, China) Ammonium molybdate tetrahydrate ((NH_4_)_6_Mo_7_O_24_·4H_2_O) and hydrochloric acid were purchased from China Shanghai Sinopharm Chemical Reagent Co., Ltd. (Shanghai, China) Potassium hydroxide (KOH; purity: ≥85%) and nickel nitrate (Ni(NO_3_)_2_·6H_2_O) were purchased from China Tianjin Damao Chemical Reagent Factory. Anhydrous ethanol (C_2_H_5_OH) was purchased from China Chengdu Cologne Chemicals Co., Ltd. (Chengdu, China) PTA hydrate and PMA hydrate were purchased from China Shanghai Macklin Biochemical Co., Ltd. All the above chemicals were of an analytical grade and were used directly without any further treatment.

### 3.2. Synthesis of NMO@NF

Preparation of NMO@NF using a hydrothermal method: Before use, the commercial bulk NF was cut into small pieces (1.0 cm × 1.5 cm) and ultrasonically treated for 10 min in a mixed solution of hydrochloric acid (12.0 mol/L), ethanol, and water (hydrochloric acid/ethanol/water = 1:1:1). After treatment, the pieces were washed three times with ethanol and pure water, and the surface moisture was absorbed with filter paper. A piece of pretreated NF was placed in a 25.0 mL polytetrafluoroethylene reaction kettle containing 7.5 mL water. Subsequently, ammonium molybdate tetrahydrate (0.1125 mmol, 139.0 mg) and nickel nitrate hexahydrate (0.2625 mmol, 76.0 mg) were sequentially dissolved, and were ultrasonically dissolved for 30 min. The reaction kettle was heated in an oven at 150 °C for 6 h. After being cooled naturally to room temperature, the resulting NMO@NF was removed, washed three times with ethanol and deionized water, and finally dried at 60 °C for 10 h [39].

### 3.3. Synthesis of PMA/NMO@NF, PTA/NMO@NF, and PTA&PMA/NMO@NF

Preparation of PMA/NMO@NF, PTA/NMO@NF, and PTA&PMA/NMO@NF: The PMA/NMO@NF, PTA/NMO@NF, and PTA&PMA/NMO@NF sample was synthesized through a simple wet chemical etching process. Before soaking, PMA (0.04 mmol, 73.0 mg) was dissolved in an 8.0 mL mixture solution of ethanol and deionized water (ethanol/water = 2:1) to prepare solution A; PTA (0.04 mmol, 115.2 mg) was dissolved in an 8.0 mL mixture solution of ethanol and deionized water (ethanol/water = 2:1) to prepare solution B; and PMA (0.008 mmol, 14.6 mg) and PTA (0.032 mmol, 92.2 mg) were dissolved in an 8.0 mL mixture solution of ethanol and deionized water (ethanol/water = 2:1) to prepare solution C. Then, the NMO@NF precursor was separately soaked in the above mixed solution for 15 min; the obtained PMA/NMO@NF, PTA/NMO@NF, and PTA&PMA/NMO@NF were washed three times with ethanol and deionized water. Finally, they were dried in an oven at 60 °C for 1 h.

### 3.4. Material Characterization

The surface microstructure of the synthesized catalyst was studied using a high-resolution transmission electron microscope (HR-TEM; FEI Talos F200S, Thermo Fisher Scientific, New York, NY, USA) with a field emission gun. Additionally, the surface morphology and microstructure of the catalyst were investigated using a scanning electron microscope (SEM; SUPRA 55 Sapphire, Tokyo JEOL Ltd., Tokyo, Japan), an X-ray diffractometer (XRD; Mini Flex 600, Tokyo Rigaku Corporation, Tokyo, Japan), and a Raman spectrometer. The cation content of the samples was detected using an inductively coupled plasma optical emission spectrometer (ICP-OES; Agilent 5110, Agilent Technologies Inc., Santa Clara, CA, USA). The elemental composition and valence states of the catalyst were studied using X-ray photoelectron spectroscopy (XPS; Thermo Scientific K-Alpha, Thermo Fisher Scientific, New York, NY, USA), with the binding energy of the samples being calibrated using the C-C peak (284.8 eV) of the C 1s orbital. Elemental spectra and mapping were obtained using an energy-dispersive spectrometer (Bruker SuperX, Thermo Fisher Scientific, New York, NY, USA). Furthermore, the FEI Talos F200S transmission electron microscope was used to explore the microscope images, elemental mapping, and linear scanning analysis.

### 3.5. Electrochemical Characterization

All electrochemical data were obtained using a CHI 660E electrochemical workstation (CH Instruments Inc., Shanghai, China) equipped with a three-electrode system. The prepared NMO-based catalyst was loaded onto NF as the working electrode, with the Hg/HgO electrode and graphite electrode serving as the reference and counter electrodes, respectively. In the electrochemical tests involved in this study, the geometric area of the working electrode immersed in the electrolyte was 1.0 cm × 1.0 cm. According to Equation (1), all potentials tested under the Hg/HgO electrode were calibrated to the potential under the reversible hydrogen electrode (RHE):

E (vs. RHE) = E (vs. Hg/HgO) + 0.098 V + 0.059 pH(1)

In a 1 M of KOH solution, an OER test was conducted using linear sweep voltammetry (LSV) at a scan rate of 5 mV/s within the voltage range of 0 to 1.0 V. The Tafel slope of the samples was linearly fitted according to the Tafel equation (η = b log j + a, where η is the overpotential, b is the Tafel slope, and j is the current density). Electrochemical impedance spectroscopy (EIS) was used to determine the electrolyte resistance (R_s_) and charge transfer resistance (R_ct_) for different catalysts in the frequency range of 10 kHz to 0.01 Hz. The double-layer capacitance (C_dl_) was obtained from cyclic voltammograms (CVs) at scan rates of 10, 15, 20, 25, and 30 mV/s within the non-Faradaic current region. The 24 h stability was measured using chronoamperometry. All reported current densities were adjusted to account for ohmic potential drop, and reverse scans were conducted to avoid the effects of redox currents.

## 4. Conclusions

In summary, PTA&PMA/NMO@NF was successfully prepared via an easy and accessible hydrothermal process, followed by chemical etching with PTA and PMA. The obtained catalyst exhibited superior electrocatalytic effects compared to NMO@NF in OER. For the OER in 1.0 M of KOH, PTA&PMA/NMO@NF achieved a low overpotential of 200 mV at a current density of 10 mA·cm^−2^. After a 24 h current time test, the electrode maintained good stability, and the overpotential remained unchanged. The excellent performance of PTA&PMA/NMO@NF is attributed to the etching of PTA and PMA. For instance, PTA and PMA can result in the defect of the two cations (Ni and Mo). Moreover, the narrowing of the lattice structure improves the charge transfer efficiency, thereby reducing the charge transfer electrons. These changes are beneficial to electron transfer in the OER process. Moreover, the increase in the valence state of Ni ions is beneficial to the generation of NiOOH in the OER process. In addition, (PO_4_)^3−^ is attached to the NMO surface, thereby regulating the electronic configuration of the two cations (Ni and Mo). This work presents low-cost, simple, and convenient synthetic strategies to improve the catalytic activity of NMO. It also offers potential avenues for designing new materials. 

## Data Availability

The data presented in this study are available on request from the corresponding author.

## Supplementary Materials

The following supporting information can be downloaded at: https://www.mdpi.com/article/10.3390/ijms26073107/s1.
