# Supplementary material for: Polyoxometalate Etching of NMO@NF for Highly Efficient Oxygen Evolution Reaction in Water Splitting
Source: Int J Mol Sci. 2025 Mar 28;26(7):3107. doi: 10.3390/ijms26073107 (PMC11988611; doi:10.3390/ijms26073107)
Supplement: Supplementary file 1 [file ijms-26-03107-s001.zip › ijms-3513520-supplementary.pdf]

Polyoxometalate etching of NMO@NF for Highly Efficient Oxygen Evolution Reaction in Water Splitting

Ting Chen [a], Xiang Han [a], Zefen Wang [b], Chaoying Li [a], Mei Li [a], Xiongdiao Lan [a,*], Yingying Ning [a], Jingxin Wang [a], Pengru Liu [a,*]

a Guangxi Key Laboratory for Polysaccharide Materials and Modifications, School of Chemistry and Chemical Engineering, Guangxi Minzu University, Nanning 530006, China.

b Guangxi Autonomous Region Center for Analysis and Test Research, Nanning 530022, China

* Correspondence: lanxiongdiao@gxmzu.edu.cn; liu-pengru@gxmzu.edu.cn

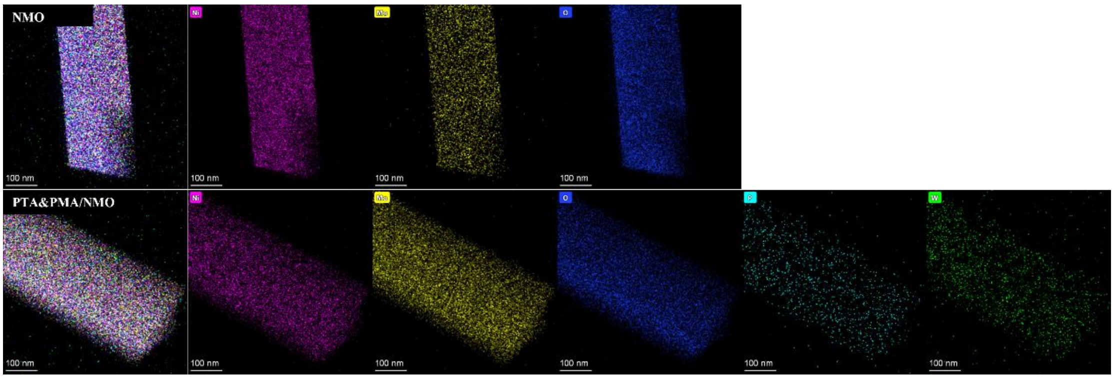

Figure S1. EDX elemental mappings of NMO, PTA&PMA/NMO

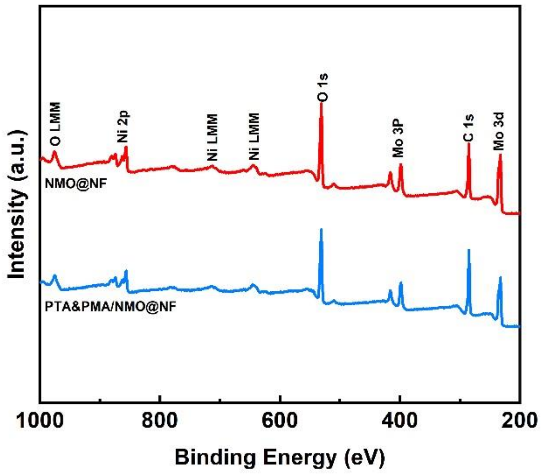

Figure S2 XPS spectra of NMO@NF and PTA&PMA/NMO@NF

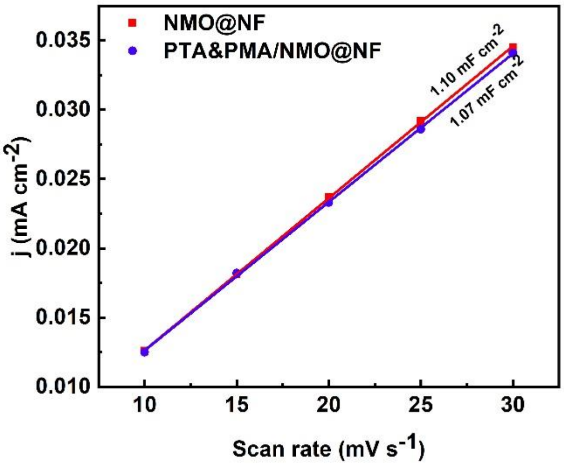

Figure S3 C_{dl} obtained by CV curves at −0.3 V versus Hg/HgO

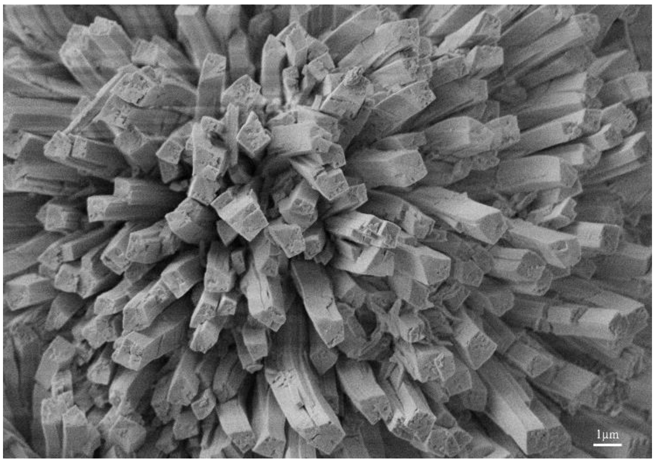

Figure S4 SEM image of PTA&PMA/NMO@NF Sat.

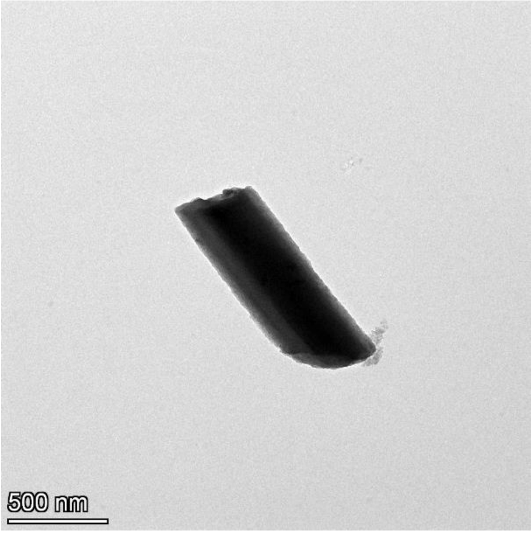

Figure S5 TEM image of NMO@NF/PTA&PMA Sat.

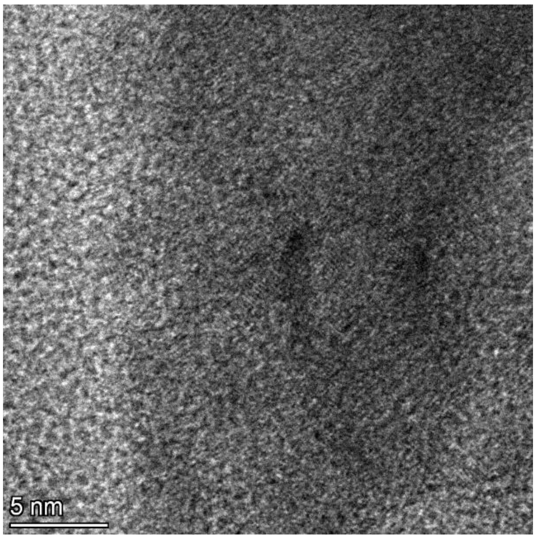

Figure S6 HRTEM image of PTA&PMA/NMO Sat.

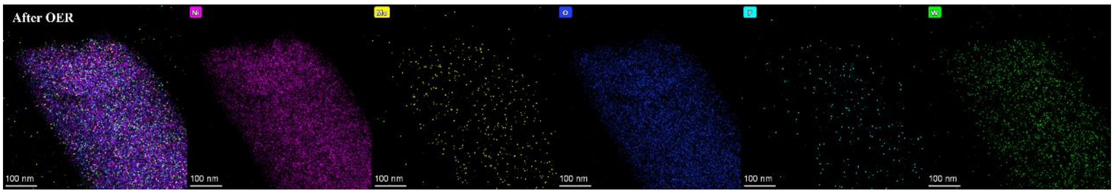

Figure S7 EDX elemental mappings of PTA&PMA/NMO Sat.

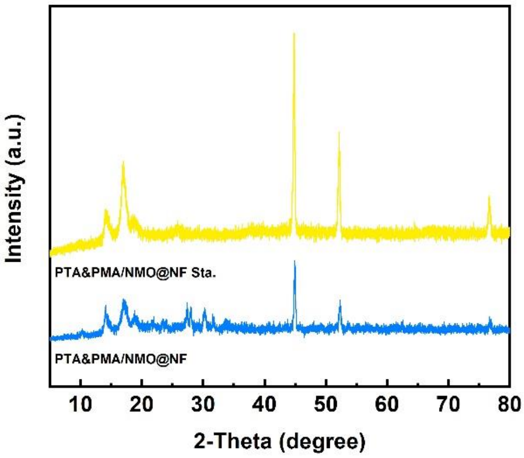

Figure S8 XRD pattern of PTA&PMA/NMO@NF before and after OER

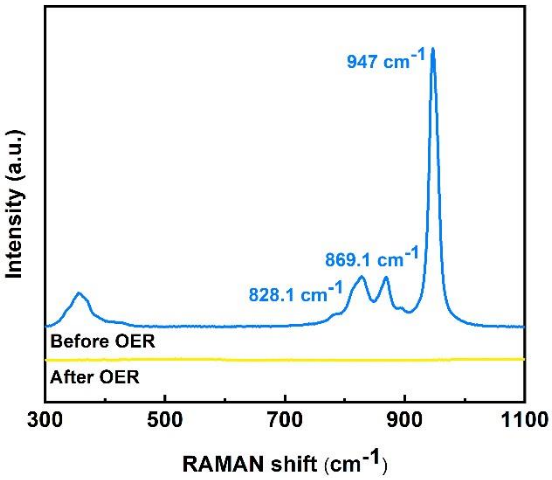

Figure S9 Raman pattern of PTA&PMA/NMO before and after OER

Table S1 ICP-OES resuits of NMO, PTA&PMA/NMO and PTA&PMA/NMO Sat.

**Sample**	**The content of Ni (%)**	**The content of Mo (%)**	**The content of p (μg/g)**	**The content of W (μg/g)**	**The ratio Of Ni:Mo**
NMO	28.87	46.13	-	-	0.626
PTA&PMA /NMO	25.94	45.36	280.7	67.3	0.572
PTA&PMA /NMO Sat.	52.57	0.12	163.57	495.22	438.1

Table S2 The surface compositions of NMO, PTA&PMA/NMO@NF and PTA&PMA/NMO@NF Sat. derived from XPS test

Sample	Metal oxygen (O 1s)	O vacancies (O 1s)	Ni3+/Ni2+ (Ni 2p)
NMO@NF	17.1 at.%	1.3 at.%	0.58
PTA&PMA /NMO@NF	23.7 at.%	7.6 at.%	0.77
PTA&PMA /NMO@NF Sat.	2.95 at.%	18.4 at.%	4.50

Table S3 TEM-EDX resuits of NMO, PTA&PMA/NMO and PTA&PMA/NMO Sat.

Sample	Ni (At. %)	Mo (At. %)	O (At. %)	P (At. %)	W (At. %)
NMO	29.7	23.7	46.6	-	-
PTA&PMA /NMO	21.2	15.9	62.3	0.201	0.399
PTA&PMA /NMO Sat.	30.7	0.224	68.5	0.178	0.398

Table S4 Comparison of OER performance of catalysts in this work and other reported transition metal electrocatalysts in alkaline media.

Catalyst	Electrolyte	J (mA cm^{-2})	η (mV)	Tafel slope ($mV\ dec^{-1}$)	References
**NMO/PTA&PMA**	**1 M KOH**	**10**	**200**	**29.4**	**This work**
F-$CoMoO_{4-x}$-2@GF	1 M KOH	10	256	64.4	1
NiFe LDH-PMo12	1 M KOH	10	206	47.5	38
PNMO@Pi/NF	1 M KOH	10	260	73	39
NMO-30M	1 M KOH	10	260	85.7	42
CoMoO4-Ov-2@GF	1 M KOH	10	296	62.4	56
$CoMoO_4$ nanoparticles	3 M KOH	10	200	149	57
$FeNiS_2$	1 M KOH	10	250	99	58
$LaNiO_{2.9}F_{0.1}$	1 M KOH	10	320	78	59
